# Vitamin D supplementation to persistent carriers of MRSA—a randomized and placebo-controlled clinical trial

**DOI:** 10.1007/s10096-018-3306-7

**Published:** 2018-06-21

**Authors:** Linda Björkhem-Bergman, Catharina Missailidis, John Karlsson-Valik, Ann Tammelin, Lena Ekström, Matteo Bottai, Ulf Hammar, Gudrun Lindh, Peter Bergman

**Affiliations:** 1 Division of Clinical Geriatrics, Department of Neurobiology, Care Sciences and Society (NVS), KI Huddinge, Stockholm, Sweden; 2 Palliative Home Care and Hospice Ward, ASIH Stockholm Södra, Älvsjö, Sweden; 3 Department of Laboratory Medicine (LABMED), Clinical Microbiology, KI Huddinge, Stockholm, Sweden; 4 Södersjukhuset, Infectious Diseases Unit, Stockholm, Sweden; 5 Division of Infectious Diseases, Department of Medicine Solna (MedS), KI, Stockholm, Sweden; 6 Department of Laboratory Medicine (LABMED), Clinical Pharmacology, KI Huddinge, Stockholm, Sweden; 7 Biostatistics Unit, Institute of Environmental Medicine (IMM), KI, Stockholm, Sweden; 8 Department of Infectious Diseases, Karolinska University Hospital, Stockholm, Sweden

**Keywords:** Methicillin-resistant *S. aureus* (MRSA), Vitamin D, Clinical trial, Immunity

## Abstract

Methicillin-resistant *Staphylococcus aureus* (MRSA) is resistant to all beta-lactam antibiotics and can cause severe infections that are difficult to treat. Eradication strategies with conventional antibiotics are not always effective and alternative approaches are warranted. Here, we tested the hypothesis that daily supplementation with vitamin D for 12 months would reduce MRSA carriage rates among a group of persistent carriers. This was a double-blind, placebo-controlled randomized trial with *n* = 65 persistent MRSA carriers with 25-hydroxy vitamin D_3_ (25OHD) < 75 nmol/L, who were followed up with bacterial cultures at baseline and every 3 months for 1 year. The primary endpoint was the decline in MRSA positivity during the study period. The study was conducted in two MRSA outpatient clinics at the Karolinska University Hospital, Stockholm, Sweden. In total, *n* = 65 persistent MRSA carriers were randomized and *n* = 3 were lost to follow-up. Only patients deficient in vitamin D (< 75 nmol/L) were included. Vitamin D (4000 IU) or placebo/day was administered for 12 months. The decline in MRSA positivity was equal in the vitamin D and placebo group during the study period (OR, 1.00; 95% CI, 0.97–1.03; *p* = 0.928) and approximately 40% in both groups were MRSA-negative after 12 months. The vitamin D group produced 103 positive cultures out of 318 cultures (32.4%) from nose, throat, and perineum over the study period, whereas the placebo group produced 135/393 positive cultures (34.0%) (Fisher’s exact test, *p* = 0.94). Vitamin D supplementation did not influence MRSA carriage. Thus, available data does not support vitamin D supplementation to persistent MRSA carriers.

**Trial registration:** www.clinicaltrials.gov; NCT02178488.

**Electronic supplementary material:**

The online version of this article (10.1007/s10096-018-3306-7) contains supplementary material, which is available to authorized users.

## Introduction

Methicillin-resistant *Staphylococcus aureus* (MRSA) are resistant to beta-lactam antibiotics and cephalosporins and can cause severe infections, especially in elderly and weak patients. Historically, MRSA infections were more prevalent among patients within the health care sector (i.e., hospital-acquired or hospital-associated MRSA). Recent surveys suggest that community-acquired or community-associated onsets are becoming more common. Findings of MRSA in screening or clinical cultures are notifiable to the Public Health Agency by Swedish Communicable Disease Act [1]. Official statistics show that the number of new cases increases each year; in 2016, 4397 cases of MRSA were reported in Sweden, an increase of 12% compared to 2015 and the total amount of new notifiable cases (colonization and infection) has doubled every fifth year since 2007 [2]. Precautions have been made using ordinary infection control methods to limit the spread. In addition, all MRSA-positive cases are tracked and bacterial clones are regularly subjected to genetic analysis at clinical bacteriology laboratories in order to identify spreading patterns. Notably, clinical disease with MRSA is only the tip of the iceberg with regard to prevalence. The majority of cases never present clinical symptoms but rather constitute a reservoir for the bacterium in society. Therefore, in Stockholm County, all patients with risk factors such as skin lesions, chronic wounds, and urinary catheters are cultured for the presence of MRSA on admission to hospital. In cases of a positive culture, the patients are placed separately from other patients, in order to limit the risk of contamination and spread to other patients and hospital staff.

The population can be divided in three groups with regard to carriage of *S. aureus,* including MRSA: “never carriers,” “intermittent carriers,” and “persistent carriers” [3]. It is possible to eradicate MRSA from mucosal surfaces using a combination of topical and systemic antibiotic treatment. However, results are varying and especially persistent carriers often experience that MRSA colonization reoccurs after some time (3–6 months after eradication). Long-term antibiotic treatment has been considered to be unfeasible due to effects on the normal flora, risk of developing allergy, and also for developing resistance against the used antibiotic treatment. Notably, the reason(s) why certain individuals are persistent carriers of MRSA remain elusive.

Thus, alternative treatment strategies or preventive measures are highly warranted. Here, we hypothesized that vitamin D could eliminate MRSA carrier status among a group of persistent carriers in Stockholm, Sweden. The rationale behind our hypothesis is based on several pieces of evidence: (i) carriers of *S. aureus* have lower vitamin D levels than non-carriers [4, 5]; (ii) vitamin D induces the expression of several antimicrobial peptides (AMPs) that can kill *S. aureus*, including MRSA [6]; (iii) atopic skin, which is highly susceptible to *S. aureus* colonization has impaired expression of AMPs [7]; and (iv) results from an interventional study previously performed by us showed that vitamin D-treated patients had significantly fewer bacterial cultures that were positive for *S. aureus* [8].

To formally test the hypothesis, we performed a double-blind, randomized, and placebo-controlled clinical trial (RCT), where *n* = 65 persistent MRSA carriers were recruited and allocated to receive either placebo or 4000 IU vitamin D daily during 12 months. The primary endpoint was the decline in MRSA carriage during the study period.

## Results

### Baseline demographics—description of the cohort

**Fig. 1:**
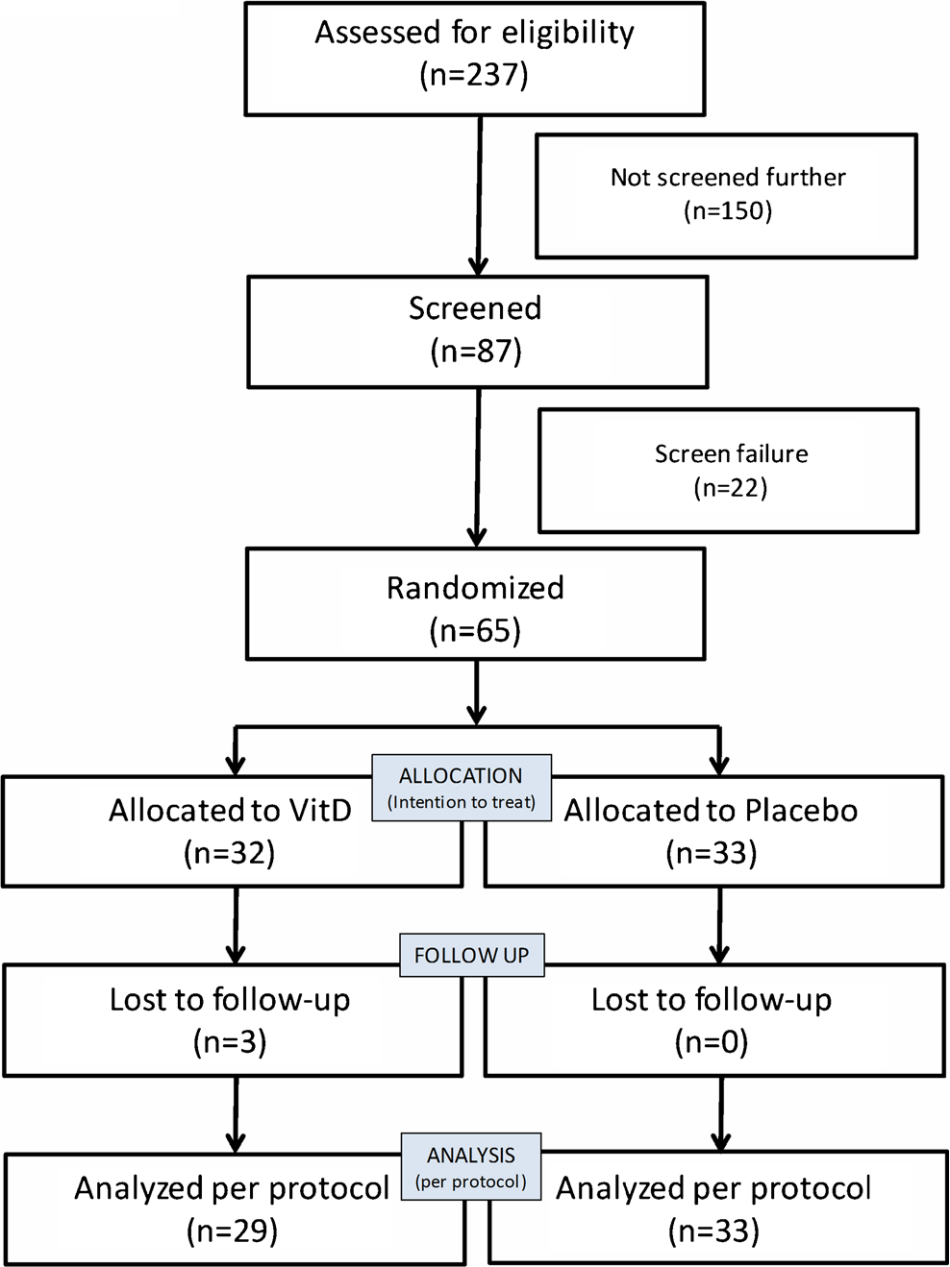
CONSORT chart

Two study sites were used for patient recruitment, the MRSA outpatient clinics at the Karolinska University Hospital in Solna and Huddinge. In total, *n* = 237 patients were assessed for eligibility, *n* = 87 were screened, and *n* = 65 were randomized to take either placebo or 4000 IU cholecalciferol per day for 12 months. The intention-to-treat (ITT) population consisted of *n* = 65 patients (vitamin D, *n* = 32; placebo, *n* = 33) and was used for the main analysis (Fig. 1). The included patients exhibited an equal distribution between the groups with regard to gender, but females were more common in both groups (placebo group, 55%; vitamin D group, 59%). Median baseline 25-hydroxy vitamin D_3_ (25OHD) levels were 49 nmol/L. The median number of years with MRSA was 3 in the placebo group and 2 in the vitamin D group (range 1–4 in both groups, *p* = 0.88). A subset of patients had risk factors for MRSA carriage (eczema, abscesses, chronic wounds, and diabetes), but these were equally distributed between the groups (Table 1). The most common location for MRSA carriage was the nose (placebo, 61%; vitamin D, 69%), followed by the throat (42 and 41%) and perineum (30 and 25%). Most individuals had MRSA at mixed locations (nose, throat, perineum); *n* = 6 were only positive for MRSA in the nose in each group, whereas *n* = 3 were positive in all three locations in the placebo group and *n* = 2 in the vitamin D group. Few individuals had 25OHD levels below 25 nmol/L (*n* = 9), but a substantial portion of included individuals had 25OHD levels below 50 nmol/L (*n* = 35) (Table 1).

**Table 1:** Baseline characteristics of included patients (ITT population)

Factor	Level	Placebo (*n* = 33)	Vitamin D (*n* = 32)
Sex	Female	18 (55%)	19 (59%)
	Male	15 (45%)	13 (41%)
BMI, median (IQR)		27.15 (24.0–31.6)	25.82 (23.6–30.6)
Age, median (IQR)		41.03 (31.3, 58.0)	41.40 (34.9–48.9)
25OHD level, median (IQR)		49.0 (37–61)	49.5 (33–63)
Height, median (m, IQR)		1.69 (1.6–1.8)	1.68 (1.6–1.7)
Weight, median (kg, IQR)		82 (67–98)	80 (65–90)
Years with MRSA, median (IQR)		3 (1–4)	2 (1–4)
Eczema	Yes	8 (24%)	6 (19%)
Abscess	Yes	4 (12%)	1 (3%)
Wound	Yes	0 (0%)	1 (3%)
Diabetes	Yes	6 (18%)	1 (3%)
Smoker	Yes	4 (12%)	2 (6%)
Have pet	Yes	7 (21%)	7 (22%)
MRSA in the family	Yes	13 (39%)	13 (41%)
Immunosuppression	Yes	2 (6%)	2 (6%)
Skin disease	Yes	6 (18%)	5 (16%)
Nose: MRSA-positive	Yes	20 (61%)	22 (69%)
Throat: MRSA-positive	Yes	14 (42%)	13 (41%)
Perineum: MRSA-positive	Yes	10 (30%)	8 (25%)
Vitamin D-deficient < 25 nmol/L	Yes	5 (15%)	4 (13%)
Vitamin D-deficient < 50 nmol/L	Yes	18 (55%)	17 (53%)

*BMI* body mass index, *IQR* interquartile range

### The primary endpoint

**Fig. 2:**
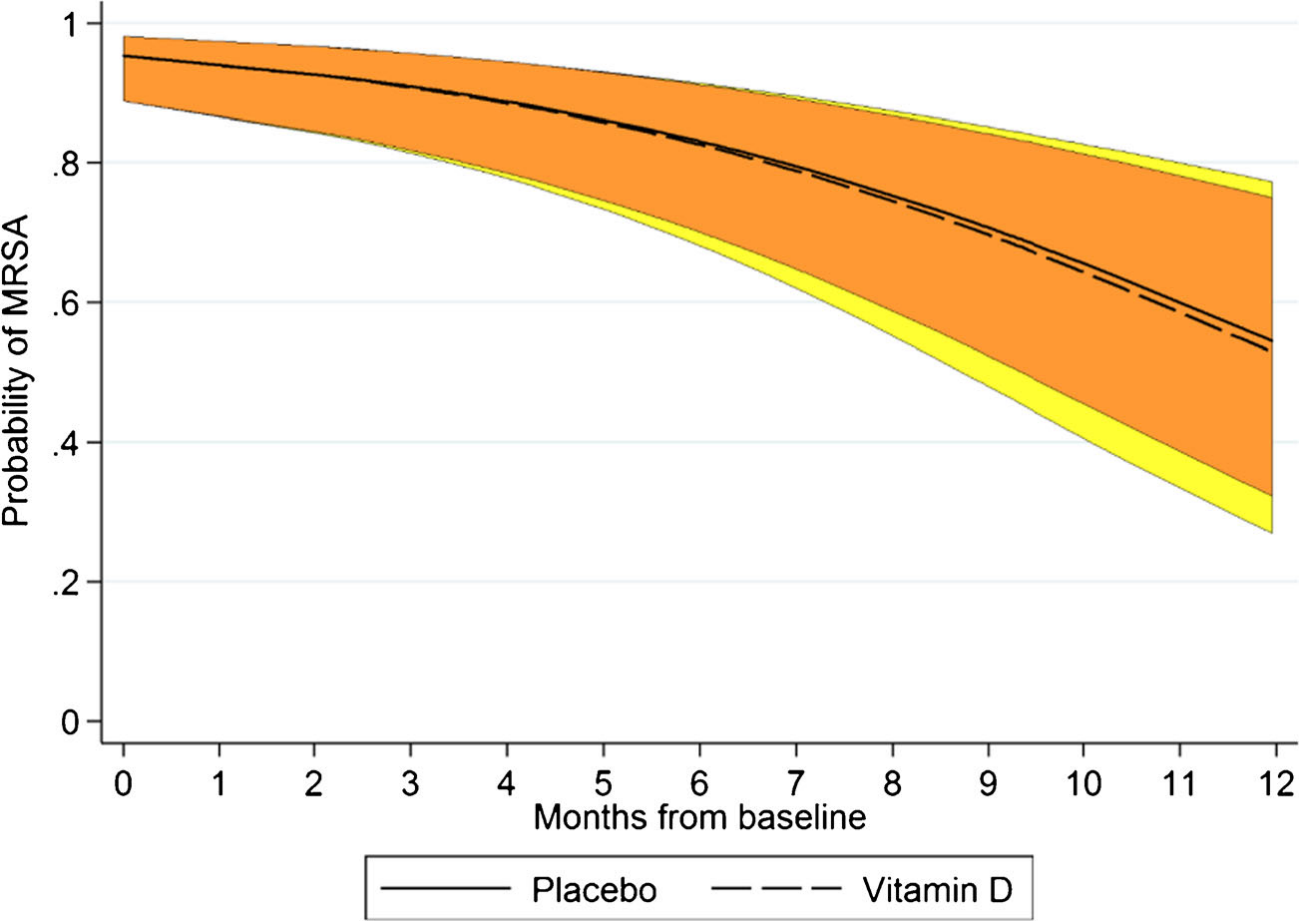
The primary endpoint was the slope of the probability to carry MRSA, based on values for baseline, 3, 6, 9, and 12 months. The model included subjects as random effect, time (in weeks) as a continuous variable, and an interaction between time and treatment group. The colored area represents the 95% confidence interval; yellow is for the vitamin D group and orange is for the placebo group

The primary endpoint was defined as the decline of MRSA positivity measured over a 12-month period. Patients were sampled for MRSA carriage every 3 months at three sites (nose, throat, and perineum). A positive culture at any site was defined as being MRSA-positive. There was no significant interaction between treatment group and time (Fig. 2, *p* = 0.928), with approximately 40% of randomized individuals being negative after 12 months with treatment. The fraction of positive individuals was not significantly different between the groups at any time during the study (placebo, 64% positive; vitamin D, 52% positive at end of treatment period after 12 months, *p* = 0.42, Fisher’s exact test, Table 2). The vitamin D group produced 318 cultures from nose, throat, and perineum over the study period and 103 were positive (32.4%), whereas the placebo group produced 393 cultures and 135 were positive (34.0%) (Fisher’s exact test, *p* = 0.94).

**Table 2:** The fraction of MRSA-positive individuals at each time point

	Baseline	3 months	6 months	9 months	12 months
Placebo	33/33 (100%)	22/32 (69%)	23/33 (70%)	17/33 (52%)	21/33 (64%)
Vitamin D	32/32 (100%)	20/29 (69%)	18/28 (64%)	16/24 (67%)	13/25 (52%)
*p* value	1.00	1.00	0.79	0.24	0.43

The figures designate numbers of individuals being positive for MRSA, divided per total number of individuals with available information. The percentage is written out in parenthesis

### Secondary endpoint—vitamin D levels in serum

**Fig. 3:**
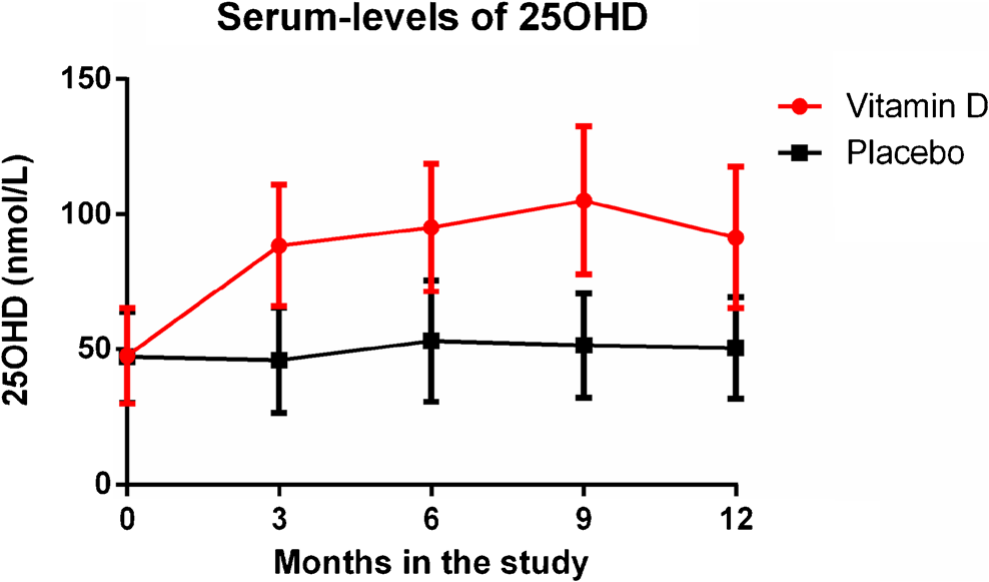
Serum levels of 25-OH vitamin D during the study. Error bars depict standard error of the mean (SEM)

The 25OHD level in serum was a predefined secondary endpoint. Median levels of 25OHD remained around 50 nmol/L in the placebo group during the study period, whereas the vitamin D group increased already after 3 months and reached levels around 80–100 nmol/L that were sustained during the study (Fig. 3). Combined, these results demonstrated a good compliance to the study protocol.

### Subgroup analysis—was the intervention efficient in any subgroup?

**Fig. 4:**
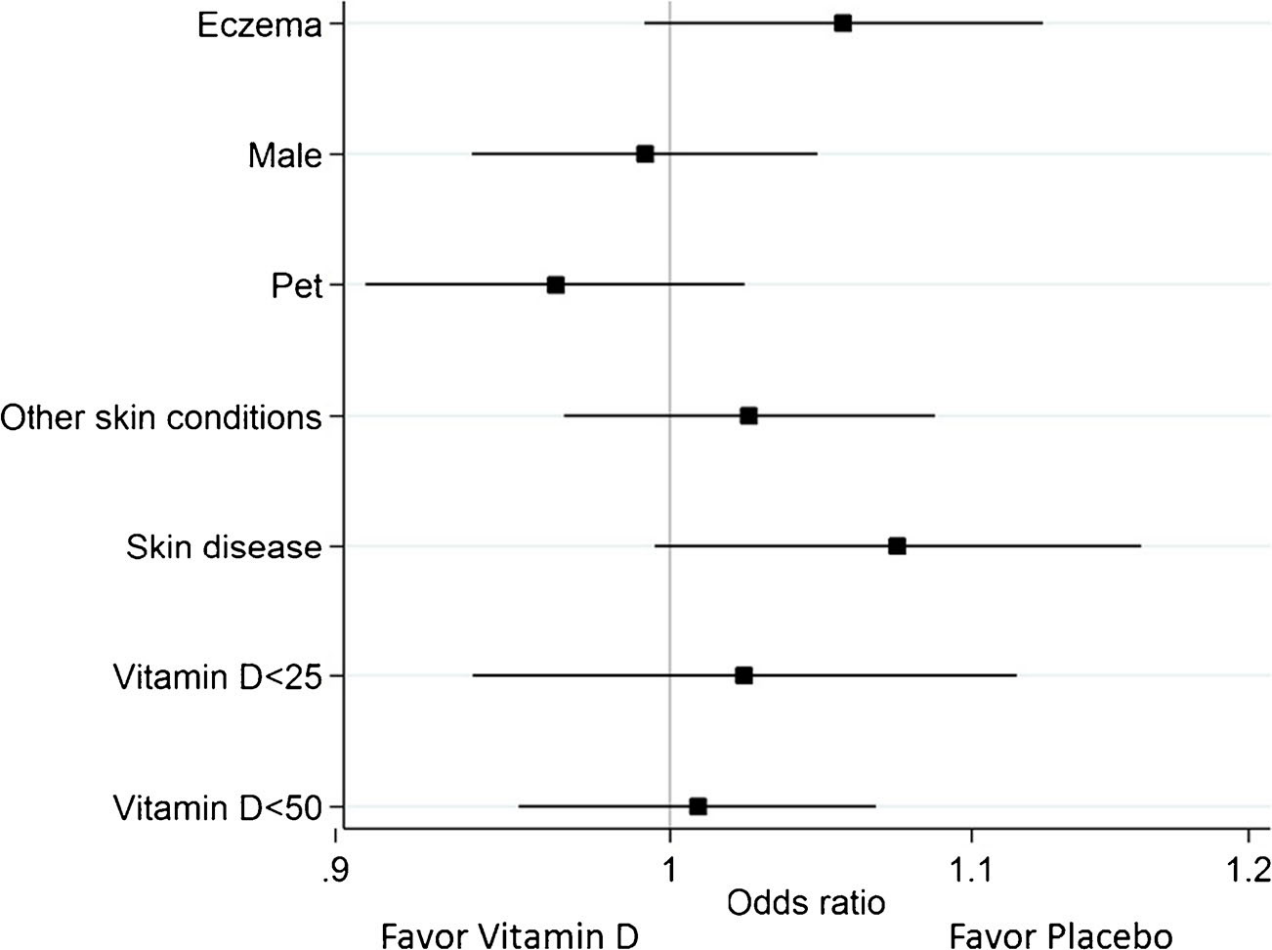
The effect of vitamin D supplementation in different subgroups was tested by interaction-analysis between time, allocation, and various pre-specified risk factors for MRSA carriage. The results are presented in a forest plot where the square represents the point estimate and the line is the 95% confidence interval. If the line crosses the perpendicular line for OR = 1.0, the effect is not significant

Since previous studies have shown that vitamin D supplementation is more effective in vitamin D-deficient individuals, we performed various subgroup analyses, including study participants below 25 and 50 nmol/L, respectively. Notably, there was no significant three-way interaction between baseline 25OHD levels, treatment, and time (OR 1.02, *p* for interaction < 25 = 0.59, OR 1.01, *p* for interaction < 50 = 0.63) (Fig. 4). In addition, we performed similar subgroup analyses for other factors of potential interest in relation to MRSA carriage (eczema, gender, pet in the home, skin disease, and smoking) and found no significant interactions (Fig. 4). Finally, we studied the role of vitamin D-related genotypes, CYP24A1, CYP2R1, VDR (Fok1, Taq1), and GC, and could not observe any significant impact of these polymorphisms in relation to the study outcome (data not shown).

### Post hoc analysis of factors associated with MRSA carriage after 12 months of treatment

We could not observe an effect of the predefined intervention (vitamin D) on the main outcome. However, approximately 40% of study participants were MRSA-negative after 12 months of treatment, independent of the allocation. Thus, we analyzed other factors for potential association with clearing MRSA from the nose, throat, or perineum. As expected, the use of anti-MRSA antibiotics was associated with a negative MRSA culture after 12 months (OR 0.033, 95% CI 0.002–0.774). No such association was found with the use of antibiotics lacking effect against MRSA (OR 0.484, 95% CI 0.098–2.394, *p* = 0.374). In addition, no other of the predefined parameters (presence of eczema, pet in the home, concomitant skin disease, smoking, or genetic polymorphisms) were associated with negative MRSA cultures after 12 months (data not shown).

**Fig. 5:**
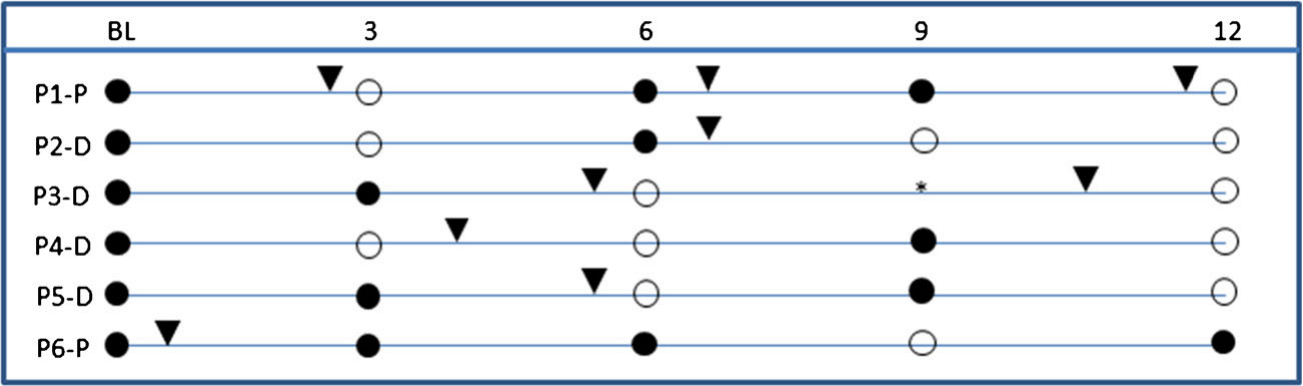
*N* = 6 study participants that were given anti-MRSA antibiotics during the study period are depicted as P1–P6, followed by a “P” for placebo and “D” for vitamin D. The patients were sampled for bacterial cultures (nose, throat, and perineum) for every 3 months from baseline until 12 months. MRSA-positive cultures are shown as filled circles, MRSA-negative cultures are shown as open circles, and anti-MRSA antibiotics are shown as black triangles

Six individuals were treated with various antibiotics (Bactrim, Dalacin, and Bactroban) during the study and five of these were negative after 12 months. One individual was initially cleared from MRSA, but the bacterium reappeared and a second treatment was needed to obtain negative cultures. Notably, one individual obtained MRSA antibiotics and cleared from MRSA but turned out to be positive again after 12 months, further underlining the fact that MRSA eradication is possible but that relapses may occur (Fig. 5).

### Adverse events

The adverse events were few and consisted of five events in the vitamin D group and two events in the placebo group. The symptoms included dry cough, nausea, fever of unknown cause, tooth extraction, and three events of spinal surgery. The adverse reactions were considered to be unrelated to the study drug in all cases.

## Discussion

Here, we report the results from a randomized, placebo-controlled trial where we have tested the hypothesis that vitamin D supplementation could reduce MRSA carriage in a group of persistent carriers. The results showed no effect of 12-month intervention with 4000 IU vitamin D/day on MRSA carriage in this group of individuals. A subgroup analysis for baseline vitamin D levels was performed but did not reveal any significant interaction between baseline 25OHD levels and the primary endpoint. Other subgroup analyses were performed on complicating factors for MRSA carriage, including presence of eczema, pet in the home, concomitant skin disease, smoking, as well as five different genetic polymorphisms, without any significant interaction terms. Importantly, reported adverse events were few and judged as not being related to the study drug. The compliance in the study was excellent, as shown by increased 25OHD levels in the treatment group versus virtually non-changed 25OHD levels in the placebo group.

Results from previous observational and experimental studies provided evidence that formed the basis for the current trial. There are several reports in favor of a beneficial effect of vitamin D on MRSA carriage: (1) vitamin D is known to produce AMPs with anti-staphylococcal effects [6]; (2) MRSA carriers have lower 25OHD levels in serum than non-carriers [4, 5]; (3) results from our own RCT from 2012 showed that vitamin D-treated patients produced significantly fewer respiratory cultures with *S. aureus* than the placebo group did [8]; (4) a recent small study from Thailand (*n* = 20) reported that 2000 IU vitamin D/day reduced skin colonization in atopic dermatitis [10]. On the other hand, there are also data refuting a role for vitamin D in *S. aureus* carriage: (1) it is known that the LL-37, which is the major AMP being produced by vitamin D, requires quite high concentrations and a specific ionic environment to be active against *S. aureus* [11]; (2) an observational study in diabetic patients found no connection between 25OHD levels in plasma an *S. aureus* carriage [12]; (3) and finally, a large RCT from New Zealand including *n* = 322 healthy adults who were given monthly bolus doses of 100,000 IU failed to find an effect against *S. aureus* carriage [13].

This study has several strengths. First, we had access to a unique cohort of persistent MRSA carriers in the Stockholm County, Sweden, an area where all patients who are MRSA-positive are registered and followed up closely. The discovery of MRSA positivity most often occurs on admission to a hospital, since all risk factors, such as wounds, eczema, and catheters, are screened for MRSA. This arrangement in Stockholm enabled us to perform the current study, where we could strictly include persistent carriers, here defined as two MRSA-positive bacterial cultures from at least one location, at least 3 months apart and during 3 years prior to inclusion. Second, since baseline levels of 25OHD have been shown to be important for the effects of vitamin D supplementation, we only included patients with < 75 nmol/L. Finally, the study was a randomized, double-blind, and placebo-controlled study and the compliance was excellent with a clear increase of serum 25OHD levels in the vitamin D arm but not in the placebo group.

Along with its strengths, the study has several weaknesses, which need to be taken into account. First, and most important, the study comprised only *n* = 65 patients due to early termination of inclusion and was therefore underpowered. There were several reasons for the early termination of the study, including a major reorganization in the Stockholm health care system for MRSA carriers, slow inclusion rate, lack of staff, lack of eligible patients, and due to short duration date of the study drug. The new power calculation revealed that the power decreased from 80.2% down to 40.4% with an expected difference between intervention and placebo of 12%. This low power could lead to the fact that a true beneficial effect of vitamin D would be missed (type 2 error). Nevertheless, we performed stringent statistical analyses of the whole dataset and failed to produce any significant effects or any trends of beneficial effects. It should be noted that the study protocol included sampling at baseline and after every 3 months during the first year. In total, the vitamin D arm produced 318 cultures from nose, throat, and perineum over the study period, whereas the placebo group produced 393 cultures, which when combined provided a large dataset. The fraction of positive cultures was almost identical in the two groups (vitamin D, 32.4%; placebo, 34%; Fisher’s exact test, *p* = 0.94), which further lend credibility to the overall null result. Combined, it is not likely that a larger study would produce a different result.

Another problem was that there were few individuals with 25OHD levels < 25 nmol/L. It appears that the best effect of vitamin D supplementation occurs in individuals below this level [14]. Apparently, persistent MRSA carriers in the Stockholm area have reasonably good 25OHD status with a median level of 50 nmol/L, which is in line with another cohort of non-MRSA carriers in Stockholm with 49.5 nmol/L [8]. A strict inclusion criteria of < 25 nmol/l could have been used but the study should then have been carried out in another setting where vitamin D deficiency is more common.

The only parameter that was significantly associated with a negative carrier status after 12 months was the use of antibiotics with activity against MRSA but not for other antibiotics. Although this finding was expected, the eradication strategy was not always effective, since only 5/6 individuals cleared MRSA after antibiotic treatment. Thus, there are strong indications that also other factors are involved in MRSA carriage. For example, there could be intracellular reservoirs for the bacteria, protecting against antibiotics with poor intracellular penetrance [15]. In addition, host genetic factors could be involved. In fact, polymorphisms in a vitamin D-related gene have been shown to be associated with MRSA carriage [16]. Notably, no such effect could be found in the current work, but the sample size hampers drawing definite conclusions. Genome-wide association studies (GWASs) for carriage in humans identified SNPs in IL4, DEFB1, CRP, and VDR for persistent nasal carriage [17]. More recently, the composition of the microbiota was shown to play a major role in *S. aureus* carriage. For example, commensal *Corynebacteria* in the nose were shown to produce compounds with anti-*S. aureus* effects [18]. The skin commensal *Staphylococcus lugdunensis* was found to produce a novel antibacterial peptide (Lugdunin) with potent effects against *S. aureus* [19]. In addition, coagulase negative staphylococci (CoNS) were shown to produce other antimicrobial peptides that could kill *S. aureus* in human skin. These CoNS were absent in atopic skin, which contain high amount of *S. aureus* [20]. Combined, the presence of commensal staphylococci appears to protect against *S. aureus* and also—theoretically—against MRSA carriage. Despite the negative findings in this work, it is still possible that vitamin D could lead to beneficial changes on the microbiota composition (promoting commensal staphylococcal colonization) in MRSA carriers, but this needs to be investigated in a larger study, including a longer follow-up time.

The results presented here do not support the use of vitamin D supplementation to persistent MRSA carriers. Even though the study was small, it had a stringent RCT design with many sample points during a 12-month period, which lend credibility to the findings. Current eradication strategies with conventional antibiotics are still not 100% effective and MRSA carriage often reoccurs in a subset of the population. Thus, there is still an unmet medical need to find novel strategies to eradicate MRSA from mucosal surfaces. Future studies could involve modulation of the microbiota by introducing specific strains with anti-staphylococcal effects, staphylococcal vaccination, or other host-directed therapies in combination with conventional antibiotics.

## Materials and methods

### Ethical statement and registration

The study was approved by the local Ethical Committee (2014/476-31/4) and the Swedish Medical Product Agency and was performed in accordance with the declaration of Helsinki. Written informed consent was obtained from all study participants. The EudraCT number is 2014-000149-53. The full protocol is available from the corresponding author upon request. The study was registered at www.clinicaltrials.gov prior to inclusion of the first patient (NCT02178488).

### Study design

This was a double-blind, randomized, and placebo-controlled trial design. Patients were given the study drug (4000 IU vitamin D or placebo daily) during a 12-month period and were followed up closely during this period with visits every 3 months. After the first 12 months, the intervention was stopped and a predefined interim analysis was performed (reported here).

### Study population

At the time of the study, all MRSA carriers in Stockholm (*n* = approximately 2000) were listed at the Infectious Disease Clinic, Karolinska University Hospital and were considered as eligible. Patients were recruited from December 2014 until December 2015.

### Inclusion criteria

Persistent MRSA carriers is defined as two MRSA-positive bacterial cultures from at least one location, at least 3 months apart and during 3 years prior to inclusion; men and women aged ≥ 18–75; signed informed consent; negative pregnancy test (U-hcg); and acceptance to use adequate contraceptive methods (oral contraceptives, hormone/copper spiral). Only patients deficient in vitamin D (< 75 nmol/L) were included.

### Exclusion criteria

Exclusion criteria are as follows: vitamin D supplementation within at least 6 months prior to inclusion; serum level of 25OHD > 75 nmol/L; ongoing and continuous antibiotic treatment 30 days prior to inclusion; known sarcoidosis; primary or secondary hyperparathyroidism; kidney failure as defined as an elevated age-adjusted creatinine; long-term systemic treatment with corticosteroids or other immunosuppressive medication; medication with thiazide diuretics; hypercalcemia; ongoing malignant disorder; plans to leave the Stockholm County within 12 months of inclusion; history of kidney stones; pregnancy (ongoing or planned); breastfeeding women; participation in another clinical study involving drugs; hypersensitivity to cholecalciferol and/or any of the excipients; and other criteria that could jeopardize the study or its intention as judged by the investigator.

### Study intervention

The study drug vitamin D_3_ 20,000 IU/ml (Oral solution, Oil) as well as the placebo drug (MygliOil) was manufactured by Merck KGaA, Germany. The study medication is approved on the Scandinavian market under the name of Detremin 20,000 IU/ml. The study medication provided contained 667 IU/drop. The drug was given per oral route and 4000 IU is equivalent to six drops that were taken each day.

### Randomization

The randomization list (block 2 × 2) was generated by an external provider (Apoteket Produktion & Laboratorium AB, APL, Stockholm, Sweden) and stored during the duration of the study. Two randomization lists were created, one for each site (Karolinska University Hospital, Solna, and Karolinska University Hospital, Huddinge). The study team (nurses, doctors, and statistician) was blinded to the allocation during the whole study period.

### Concomitant medication and restricted medication

No vitamin D substitution outside of the study was allowed. All participants were instructed to avoid systemic corticosteroids and antibiotics, but if taken for medical reasons, the information was recorded in the case record form (CRF).

### Laboratory safety assessments

The safety parameters were plasma-calcium and 25OHD, which were analyzed at all time points. The results were blinded from the study team and sent to an independent study physician for evaluation. No patient in the current study was withdrawn for safety reasons.

### Analysis of genotypes

Detailed information can be found in the supplementary method section.

### Study outcomes

The primary endpoint was defined as the decline of MRSA positivity in persistent carriers during a 12-month period in the treatment groups (vitamin D or placebo) based on five measurements with 3-month intervals. Secondary endpoints are fractions of persistent MRSA-positive carriers after 12 months and serum levels of 25OHD. The analyses were carried out by the routine clinical chemistry laboratory, Karolinska University Laboratory (LIAISON-method, DiaSorin).

### Analysis of genotypes

Genomic DNA was isolated from 200 μl peripheral blood leucocytes using the DNA Blood Mini Kit (Qiagen, Hilden, Germany). Allelic discrimination reactions were performed using approximately 20 ng of DNA and TaqMan Universal Master Mix (ThermoFisher) in 10-μl reactions. The TaqMan® genotyping assays used were all from ThermoFisher (Cat. No. 4351379) with product numbers C__2958431_10 for CYP2R1 rs20607939, C_29958084_10 for CYP24A1 rs6013897, C_26407519_10 for GC rs2282679, C_2404008_10 for VDR rs731236 (here referred to as Taq1), and C__12060045_20 for VDR rs2228570 (here referred to as Fok1). The PCR profile consisted of 95 °C for 10 min followed by 40 cycles of 92 °C for 15 s and 60 °C for 1 min. The fluorescence signal was measured with an ABI 7500 Sequence Detector (Applied Biosystems).

### Safety endpoint

The safety endpoint was the frequency of adverse events (AE) among all subjects and the levels of calcium in plasma and urine (selected patients).

### Sample size calculation

The sample size estimation was based on the null hypothesis that the probability of MRSA positivity declines at the same rate in the two treatment groups over a time of 12 months, from 100 to 80% carrier status (Supplementary Fig. 1). The alternative hypothesis was that in one of the two groups it would decline faster from 100% to (80-delta), where delta is the least clinically meaningful effect. Based on a previous clinical study from our center and clinical experience, we have chosen 12% reduction to be clinically significant (Supplementary Table 1). The power was estimated by simulating data for a binary outcome with probabilities with a within-individual random intercept generated from a normal distribution with mean 0 and standard deviation of 0.025. The data for the binary outcome were generated at four time points (3, 6, 9, and 12 months) and a random effect logistic regression model was used to obtain the specified power. The binary outcome was equal to 1 for all patients at baseline (persistent carriers). With the sample size of 250 patients (125/arm), the estimated power with a delta of 12% was 88.2%. We expected 10–15% attrition during the study and thus increased the original sample size to 150 patients/arm = 300 patients in total. Importantly, the logistic regression model was based on four time points, which significantly increased the power of the study compared to analyses after 12 months only (chi-square test).

However, due to unexpected logistical constraints and a slow inclusion rate, we had to stop the inclusion after *n* = 65 individuals. A new power calculation based on *n* = 60 individuals figure was performed. The outcome of this calculation is shown in Supplementary Table 1. Tis table shows the estimated power of a random effect logistic regression (all time points) to test the null hypothesis that the probability of MRSA infection declines at the same rate in two treatment groups over time from 100 to 90%. The alternative hypothesis is that in one of the two groups it declines faster from 100% to (90% – *δ*). The estimated power is shown in Table **1** for different values of *δ* (10%, 12%, ..., 20%). The power was estimated by simulating data for a binary outcome with probabilities shown in Fig. **1** with a within-individual random intercept generated from a normal distribution with mean 0 and standard deviation 0.025. The data for the binary outcome were generated at four time points: 3, 6, 9, and 12 months. The binary outcome was equal to 1 for all patients at baseline (0 months). In this model, the sample size was 60 patients, 30 per arm. Each patient was observed at baseline and at all subsequent time points. One thousand datasets were generated for each value of *δ*.

### Statistical analysis

The statistical analysis was based on measurements of MRSA carrier status at time points 0, 3, 6, 9, and 12 months after inclusion. The null hypothesis was that the probability of MRSA positivity declined at the same rate in both groups, whereas the alternative hypothesis was that it would decline faster in one of the groups. Based on previous publications and clinical experience, we expected a spontaneous elimination of MRSA in approximately 20% of the participants. The least clinically meaningful effect was defined as an additional 12% reduction of MRSA positivity. The level of significance was set to *p* = 0.05 and a two-sided test was used. The primary endpoint was analyzed with a random effects logistic regression model, which included all observed outcome data, and thus was used to estimate the ITT effect size [9]. The model included subjects as random effect, time (in weeks) as a continuous variable, and an interaction between time and treatment group. We also tested for three-way-interactions between the treatment group, time, and (in separate models) the variables “eczema,” “pet in the home,” “skin disease,” “smoking,” and “baseline 25OHD levels.” In addition, the fraction of MRSA-positive carriers after 12 months was analyzed with Fisher’s exact test (secondary endpoint). The sample size estimation is described in supplementary methods.

### Patient involvement

Patients did not take part in the study design. However, most participants were very positive to the study and assessed the burden of taking part as minor. This was also reflected by the excellent compliance and low frequency of adverse events. The results will be disseminated to participants via regular mail, where they also will be invited to submit questions to the study team.

## Electronic supplementary material

ESM 1 (DOCX 23 kb)

ESM 2 (DOC 217 kb)
