# Supplementary material for: Vitamin D supplementation to persistent carriers of MRSA—a randomized and placebo-controlled clinical trial
Source: Eur J Clin Microbiol Infect Dis. 2018 Jun 21;37(9):1735–44. doi: 10.1007/s10096-018-3306-7 (PMC6133039; doi:10.1007/s10096-018-3306-7)
Supplement: Supplementary file 1 — (DOCX 23 kb) [file 10096_2018_3306_MOESM1_ESM.docx]

**Supplementary figure 1**. The null hypothesis is that the probability in the two groups declines at the same rate (blue line). The alternative hypothesis is that in one of the two groups it declines faster (red line).

**Supplementary table 1**. Estimated power for different values of δ for the sample size n=65. The value δ represents the difference in the probability of MRSA infection at the end of the follow up period (12 months). The right column shows the power for the random effect logistic regression model.

| Expected change between groups (δ) | Estimated power |
| --- | --- |
| 0.10 | 0.306 |
| 0.12 | 0.404 |
| 0.14 | 0.509 |
| 0.16 | 0.596 |
| 0.18 | 0.737 |
| 0.20 | 0.816 |

**Supplementary table 2.** Distribution of genotypes in the study population

| Fok1 | CC | 12 (36%) | 9 (28%) |
| --- | --- | --- | --- |
|  | TC | 17 (52%) | 19 (59%) |
|  | TT | 4 (12%) | 2 (6%) |
| Taq1 | CC | 4 (12%) | 6 (19%) |
|  | TC | 13 (39%) | 11 (34%) |
|  | TT | 16 (48%) | 13 (41%) |
| GC | GG | 5 (15%) | 1 (3%) |
|  | GT | 17 (52%) | 10 (31%) |
|  | TT | 11 (33%) | 19 (59%) |
| CYP24A1 | AA | 3 (9%) | 3 (9%) |
|  | AT | 17 (52%) | 8 (25%) |
|  | TT | 13 (39%) | 19 (59%) |
| CYP2R1 | AA | 2 (6%) | 3 (9%) |
|  | AG | 30 (91%) | 27 (84%) |
|  | GG | 1 (3%) | 2 (6%) |
